# An Intraoperative Fluoroscopic Predictor of Successful Micra Implantation on the Right Ventricular Mid‐Septum

**DOI:** 10.1002/joa3.70307

**Published:** 2026-03-08

**Authors:** Misun Pak, Ken Kakita, Takashi Yamasaki, Tetsuhisa Hattori

**Affiliations:** ^1^ Arrhythmia Care Center Koseikai Takeda Hospital Kyoto Japan

**Keywords:** α‐loop shape appearance, Micra transcatheter pacing system, steerable delivery catheter, the right ventricular mid‐septum

## Abstract

**Background:**

The Micra transcatheter pacing system has been adopted globally. However, the procedural parameters for safe Micra implantation on the ventricular wall, particularly in the mid‐septum, remain unknown. This study aimed to identify the predictors of Micra implantation on the ventricular mid‐septum.

**Methods:**

We retrospectively analyzed patients who underwent Micra implantation and had computed tomography (CT) after implantation to confirm the device position. Patients were classified into mid‐septum and non–mid‐septum groups based on CT findings. To explore predictors associated with mid‐septal implantation, fluoroscopic parameters in the standard left anterior oblique (LAO) view were evaluated during implantation, including the presence of an α‐loop shape of the delivery catheter and the angle between the axis of the device cup and the tangent of the ventricular septum (angle θ). Logistic regression analysis was performed to identify predictors of mid‐septal Micra implantation.

**Results:**

Of 119 patients who underwent Micra implantation, 59 had CT confirmation. Among them, 31 (52.5%) had mid‐septal, 18 (30.5%) apical septal, and 10 (17.0%) free wall implantation. Since no cases involved basal septal positioning, the non–mid‐septum group consisted of apical septal and free wall implantations. The angle θ was significantly larger and the α‐loop shape of the catheter was more frequent in the mid‐septum group. However, multivariable analysis identified only the α‐loop shape as an independent predictor of mid‐septal implantation (adjusted odds ratio, 5.10; 95% CI, 1.40–20.7; *p* = 0.016).

**Conclusion:**

An α‐loop shape of the delivery catheter may serve as a practical fluoroscopic marker for successful mid‐septal Micra implantation.

## Graphical abstract

Fluoroscopic predictors of successful mid‐septal Micra implantation were evaluated using computed tomography confirmation. Among fluoroscopic parameters assessed in the left anterior oblique view, the presence of an α‐loop shape of the delivery catheter was independently associated with mid‐septal implantation. An α‐loop configuration may serve as a practical intraoperative marker for achieving mid‐septal Micra placement.

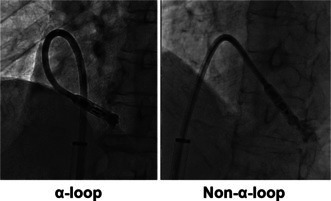

**Abbreviations**
CT: computed tomography
LAO: left anterior oblique
PICM: pacing‐induced cardiomyopathy
RA: right atrium
RAO: right anterior oblique
RV: right ventricle
TPS: transcatheter pacing system

## 1 Introduction

The Micra transcatheter pacing system (TPS), a leadless pacemaker, is widely used because it minimizes infection risk and prevents venous occlusion. Micra implantation is particularly valuable for infection prevention in patients with underlying vulnerabilities and for preserving subclavian access in those undergoing hemodialysis [1, 2]. Although early guidelines favored implantation on the ventricular apex, apical deployment is now discouraged because of associated risks such as cardiac effusion and perforation [3, 4]. Current recommendations promote implantation on the ventricular septum, particularly the mid‐septum for greater procedural safety, and mid‐septal implantation also achieves a short‐paced QRS duration [2, 5]. A shorter‐paced QRS duration can prevent patients with transvenous pacemakers from worsening heart failure and patients with a Micra implant from pacing‐induced cardiomyopathy (PICM) [6, 7].

Despite these advantages, the procedural indicators for predicting the location of Micra on the ventricular myocardium remain unclear. This study aimed to identify the procedural predictors of successful Micra implantation on the ventricular mid‐septum.

## 2 Methods

### 2.1 Study Population

This was a single‐center, retrospective, observational study. We enrolled consecutive adult patients who were implanted with Micra in our hospital between June 2018 and January 2025 on the basis of the recommendations for using leadless pacemakers of the European Society of Cardiology guidelines [8]. Micra VR or AV (Medtronic Inc., Minneapolis, MN, USA) was implanted in response to the necessity of atrial–ventricular synchrony in each patient. To analyze the location of the device in the heart, the current study included patients who underwent computed tomography (CT) imaging after Micra implantation since fluoroscopic imaging alone may be insufficient to precisely identify the actual location of the device [9]. Baseline patient information was obtained from electronic medical records. The study protocol complied with the Declaration of Helsinki and was approved by the Institutional Review Board of our hospital. The requirement for written informed consent was waived because this study employed an observational design without prespecified interventions for the study patients. However, the opt‐out option in the study protocol guarantees the right to reject enrollment.

### 2.2 Implantation Procedure

Micra implantation procedures were performed under local anesthesia with mild conscious sedation. Under ultrasound guidance, a 6 Fr sheath was inserted into the right common femoral vein. Venography through a 6 Fr sheath confirmed that the venous tract between the inferior vena cava and right atrium (RA) was patent without any stenosis, torsion, or visible thrombus. After right ventriculography using a 5 Fr Berman angiographic catheter (Teleflex, Wayne, PA, USA) through the 6 Fr sheath, the sheath was replaced with a Micra introducer sheath. The introducer sheath was advanced into the RA, and a steerable delivery catheter, including Micra, was introduced and advanced into the right ventricle (RV). The tip of the delivery catheter was securely attached to the myocardium by avoiding the septum/free wall boundary (hinge), and this was verified using contrast media in the standard right anterior oblique (RAO; 35°) and left anterior oblique (LAO; 45°) views. Following verification, the device was placed in the myocardium. Micra implantation on the ventricular basal septum was avoided because the basal septum is close to the tricuspid valve. Micra was affixed to the ventricular wall with at least two of the four nitinol tines. The tether was cut, and the delivery system was removed after ensuring the acceptable electrical parameters. Finally, biplane right ventriculography (RAO and LAO) was performed to assess the Micra position and check for cardiac effusion.

### 2.3 Identification of Predictive Factors Obtained From Fluoroscopic Imagings

**FIGURE 1:**
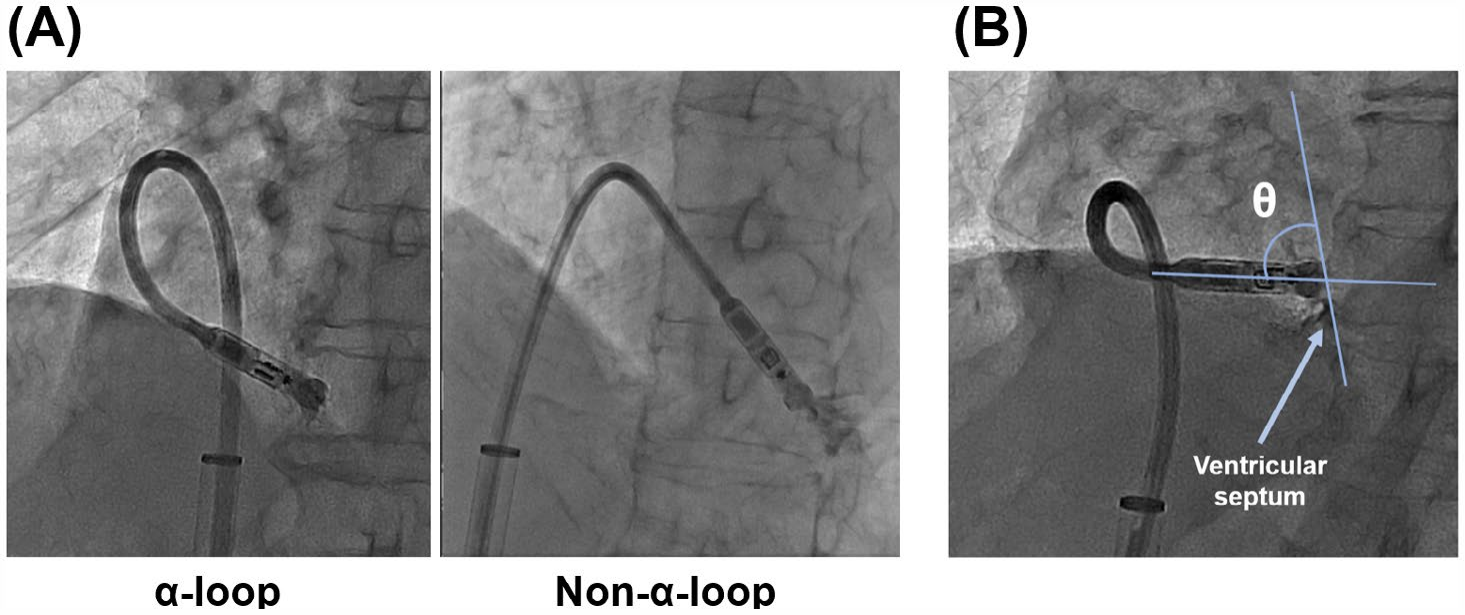
The appearance of the delivery catheter shape and the angle θ in the standard left anterior oblique (LAO) view during procedure. (A) The left figure shows the shaft of the delivery catheter forming an α‐loop, whereas the right figure does not show the delivery catheter presenting an α‐loop. (B) The angle between the axis of the device cup and the tangent of the ventricular septum just before the deployment in the LAO fluoroscopic view was defined as angle θ.

To explore procedural factors potentially associated with mid‐septal Micra implantation, specific fluoroscopic parameters were evaluated during the implantation procedure based on previous reports. A previous case report described successful mid‐septal implantation using the “α‐loop technique,” in which an α‐loop configuration of the delivery catheter is formed before deployment [10]. Additionally, near‐perpendicular contact between the catheter tip and the ventricular septum in the LAO view has been reported to increase the likelihood of septal implantation [11, 12, 13]. Accordingly, we investigated the following parameters as predictors: (1) the presence of an α‐loop shape appearance of the delivery catheter, and (2) the angle between the axis of the device cup and the tangent of the ventricular septum in the standard LAO 45° view (Figure 1A,B). During implantation, both RAO and LAO fluoroscopic projections were routinely obtained. The LAO view was used for quantitative assessment of the two predictors described above, as the LAO view is commonly employed to evaluate right ventricular septal orientation [14, 15]. In contrast, the RAO view was used as a supplemental qualitative guide in this study to assess the presence of a gooseneck sign and to estimate the distance between the catheter tip and the right ventricular septum/free wall boundary (hinge) before device deployment.

### 2.4 CT‐Based Classification of Device Location

CT imaging taken after implantation was used to determine the precise Micra implantation site. On the four‐chamber CT view, the right ventricular (RV) cavity was first divided into septal and free wall regions. The septal wall was then subdivided along the long axis into three equal segments: basal, mid, and apical septum [16]. The mid‐septum was defined as the middle one‐third of the septal wall. Using this classification, implantation sites were categorized as basal septum, mid‐septum, apical septum, or RV free wall. Statistical analyses were planned to compare clinical characteristics between the mid‐septum and non–mid‐septum groups.

### 2.5 Statistical Analysis

Continuous variables are given as average ± standard deviation or median (interquartile range [IQR; 25th to 75th percentiles]) and were analyzed using the *t*‐test or Wilcoxon rank sum test. Categorical variables were expressed as numbers and percentages and analyzed using the chi‐square test or Fisher's exact test. Logistic regression analyses were conducted to identify the predictors of Micra implantation on the ventricular mid‐septum. Univariate analyses were performed using candidate explanatory variables. Variables with *p* < 0.05 in the univariate analysis were subsequently included in a multivariate logistic regression model to determine the independent predictors. R software (package version 3.6.1, R Development Core Team) was used for all statistical analyses, and *p* < 0.05 were considered statistically significant.

## 3 Results

### 3.1 Baseline Characteristics

A total of 119 patients underwent Micra implantation at our hospital during the study period. Among them, 60 were excluded from the analysis because no CT images were obtained after implantation. Table 1 shows the baseline characteristics of the remaining 59 patients. In the entire cohort, the mean age at the time of the Micra implantation was 83 ± 8 years. Furthermore, 66.1% were male, and 31 patients (52.5%) had heart failure. Atrioventricular block was the main indication for Micra implantation (26 patients, 44.1%), followed by sick sinus syndrome (22 patients, 37.3%) and atrial fibrillation with a slow ventricular response (11 patients, 18.6%). The mean left ventricular ejection fraction was normal at 68% ± 9%.

**TABLE 1:** Baseline characteristics.

	Entire cohort (*n* = 59)	Mid‐septum (*n* = 31)	Non‐mid‐septum (*n* = 28)	*p*
Age (years)	83 ± 8	82 ± 9	84 ± 6	0.94
Sex (male, %)	39 (66.1)	18 (58.1)	21 (75.0)	0.17
Body mass index (kg/m^2^)	21.1 ± 3.6	20.2 ± 3.4	22.0 ± 3.6	0.09
Hypertension (%)	40 (67.8)	20 (64.5)	20 (71.4)	0.59
Diabetes mellitus (%)	19 (32.2)	12 (38.7)	7 (25.0)	0.26
Heart failure (%)	31 (52.5)	15 (48.4)	16 (57.1)	0.50
Previous stroke (%)	13 (22.0)	8 (25.8)	5 (17.9)	0.46
Indications for Micra implantation				
Atrioventricular block (%)	26 (44.1)	15 (48.4)	11 (39.3)	0.48
Sick sinus syndrome (%)	22 (37.3)	9 (29.0)	13 (46.4)	0.17
AF with slow ventricular response (%)	11 (18.6)	7 (22.6)	4 (14.3)	0.92
BNP (pg/mL)	241.9 (116.4–419.5)	294.4 (194.7–500.3)	177.8 (80.6–279.9)	0.06
LAD (mm)	45 ± 9	43 ± 8	47 ± 10	0.15
LVEF (%)	68 ± 9	66 ± 10	70 ± 8	0.33
RVDd (mm)	31 ± 5	30 ± 5	31 ± 6	0.54

*Note:* Categorical variables are presented as *n* (%). Continuous variables are presented as mean ± standard deviation.

Abbreviations: AF, atrial fibrillation; BNP, brain natriuretic peptide; LAD, left atrial diameter; LVEF, left ventricular ejection fraction; RVDd, right ventricular diastolic diameter.

Among the 59 patients whose Micra positions were confirmed, CT images revealed Micra implantation on the mid‐septum in 31 patients (52.5%), on the apical septum in 18 patients (30.5%) and on the ventricular free wall in 10 patients (17.0%). None of the patients had Micra implanted on the basal ventricular septum. Since no patients had Micra implantation on the basal septum, the non–mid‐septum group was composed of those with implantation on either the apical septum or the right ventricular free wall (Table 1).

### 3.2 Procedural Data, Electrical Parameters and the Paced QRS Duration

Procedural data and the electrical parameters were compared between the mid‐septum and non‐mid‐septum groups (Table 2). There were no significant differences in the parameters between the two groups, except for the angle between the axis of the device cup and tangent of the ventricular septum (defined as angle θ) and the delivery catheter shape appearance during implantation. The catheter shape appearance could not be evaluated in one patient in the mid‐septum group and three patients in the non‐mid‐septum group due to the absence of LAO fluoroscopic image. The mean angle θ was statistically larger in the mid‐septum group than in the non‐mid‐septum group (54° ± 23° vs. 38° ± 26° [*p* = 0.049], respectively), and the proportion of an α‐loop shape appearance was higher in the mid‐septum group than in the non‐mid‐septum group (24 patients [77.4%] vs. 9 patients [32.1%] [*p* < 0.001], respectively). Regarding procedural complications, cardiac effusion occurred in one patient in the mid‐septum group (3.2%), whereas no complications occurred in the non‐mid‐septum group. The risk did not differ significantly between the two groups (*p* = 0.33).

**TABLE 2:** Procedural information and electrical parameters of the Micra between the ventricular mid‐septum and non‐mid‐septum groups.

	Mid‐septum (*n* = 31)	Non‐mid‐septum (*n* = 28)	*p*
Type of device			
Micra VR (%)	17 (54.8)	16 (57.1)	0.86
Micra AV (%)	14 (45.2)	12 (42.9)	0.86
Operation time (minute)	49.1 ± 17.8	53.4 ± 17.7	0.31
Number of deployments	1.8 ± 1.2	1.8 ± 1.3	0.96
Angle θ (°)	54 ± 23	38 ± 26	0.049
Delivery catheter shape			
α‐loop shape (%)	24 (77.4)	9 (32.1)	< 0.001
Non‐α‐loop shape (%)	6 (19.3)	16 (57.1)	< 0.001
Complications			
Cardiac effusion (%)	1 (3.2)	0 (0.0)	0.33
R wave amplitude (mV)	6.1 ± 2.6	7.5 ± 4.3	0.37
Pacing threshold (V/0.24 ms)	0.95 ± 0.67	0.90 ± 0.62	0.92
Impedance (ohm)	732 ± 188	696 ± 160	0.50
Paced QRS duration (ms)	138 ± 18	160 ± 14	< 0.001

*Note:* Categorical variables are presented as *n* (%). Continuous variables are presented as mean ± standard deviation.

No significant differences were observed in the device parameters between the two groups. On the other hand, the paced QRS duration was statistically shorter in the mid‐septum group than in the non‐mid‐septum group (138 ± 18 ms vs. 160 ± 14 ms, *p* < 0.001).

### 3.3 Predictors of Micra Implantations on the Right Ventricular Mid‐Septum

A univariable logistic regression analysis revealed that both the α‐loop shape appearance of the delivery catheter (odds ratio [OR], 7.11; 95% confidence interval [CI], 2.22–25.6; *p* = 0.001) and the angle θ (OR, 0.97; 95% CI, 0.95–0.99; *p* = 0.039) might be associated with mid‐septal Micra implantation (Table 3). However, a multivariable logistic regression analysis identified that only the α‐loop shape appearance remained a significant predictor of mid‐septal implantation (adjusted OR, 5.10; 95% CI, 1.40–20.7; *p* = 0.016). The angle θ was no longer a significant predictor (adjusted OR, 0.98; 95% CI, 0.95–1.01; *p* = 0.18).

**TABLE 3:** Univariable and multivariable logistic regression analysis for predicting mid‐septal Micra implantation.

	Univariable			Multivariable		
	OR	95% CI	*p*	Adjusted OR	95% CI	*p*
Age	1.02	0.96–1.10	0.49	—	—	—
Sex category (male)	2.17	0.73–6.88	0.17	—	—	—
BMI	1.16	0.99–1.37	0.06	—	—	—
LVEF	1.05	0.99–1.13	0.15	—	—	—
LAD	1.05	0.99–1.12	0.13	—	—	—
RVDd	1.05	0.94–1.19	0.48	—	—	—
α‐loop shape of a delivery catheter	7.11	2.22–25.6	0.001	5.10	1.40–20.7	0.016
Angle θ	0.97	0.95–0.99	0.039	0.98	0.95–1.01	0.18

Abbreviations: BMI, body mass index; CI, confidence interval; LAD, left atrial diameter; LVEF, left ventricular ejection fraction; OR, odds ratio; RVDd, right ventricular diastolic diameter.

## 4 Discussion

In this study, we analyzed the fluoroscopic predictors of mid‐septal Micra deployment obtained during the procedure in patients whose Micra position was confirmed using CT imaging after implantation. A multivariable analysis that includes potential factors identified that only the α‐loop shape of the delivery catheter, which was observed just before deployment in the standard LAO view, was an independent predictor of mid‐septal implantation.

### 4.1 Association of the Appearance of the Catheter Shape Just Before Deployment With the Predictor of Mid‐Septal Implantation

The Micra TPS can be affixed to the ventricular wall by pressing the delivery catheter tip against the myocardium under appropriate pressure before deployment. Vertical contact between the catheter tip and the ventricular septum is ideal for successful device implantation on the septum. The gooseneck shape of the delivery catheter is a reliable indicator of proper contact with the myocardium. However, this does not necessarily imply that the catheter tip is positioned against the septum. Although the conventional gooseneck‐shaped technique has been widely adopted, anatomical malformations or skeletal deformities significantly hinder its application [11].

Several alternative techniques have recently been reported for deploying Micra to the ventricular septum [10, 11, 12]. Among them, the α‐loop technique has demonstrated effectiveness for mid‐septal Micra implantations [10]. This technique stabilizes the catheter tip on the mid‐septum by adjusting the rotational torque of the delivery catheter (either clockwise or counterclockwise) while applying gentle pressure against the ventricular septum. This approach facilitates the near‐perpendicular implantation of the Micra on the mid‐septum, thus leading to the formation of a distinctive α‐loop shape that is visible under LAO fluoroscopy [10, 13]. However, the α‐loop technique can be affected by the anatomical characteristics of the RV, which require adequate space to form the α‐loop shape of the delivery catheter. Creating an α‐loop shape in the apex is challenging because the apical space is smaller than the middle or basal space. Therefore, an α‐loop shape in the LAO view can imply that the Micra tends to be inevitably implanted on the ventricular mid‐septum.

Although the angle between the catheter tip axis and the right ventricular septal surface (angle θ) showed statistically significant differences between the mid‐septum and non‐mid‐septum groups, it did not significantly predict mid‐septal Micra implantation. On the basis of previous studies suggesting that the near‐perpendicular contact of the catheter tip in the LAO view increases the likelihood of septal implantation [11, 13], we hypothesized that angle θ could serve as a predictor for mid‐septal Micra implantation. However, the multivariable analysis in the current study did not support this hypothesis. This may be due to interindividual differences in the cardiac orientation, which could result in the misalignment of the standard LAO view with the actual cardiac axis. A fixed fluoroscopic angle may not provide an optimal measurement of angle θ in all cases. Tailoring the fluoroscopic angle to each patient's cardiac anatomy may improve the accuracy of angle θ as a predictive marker. Further investigation is warranted to confirm this hypothesis.

Despite its association with mid‐septal implantation, an α‐loop configuration was observed in 32.1% of cases that were ultimately classified as non–mid‐septal on CT imaging. This finding indicates that the presence of an α‐loop alone does not guarantee true mid‐septal deployment. One possible explanation is that, even when an α‐loop is observed in the LAO view, the tip of the delivery catheter may advance beyond the mid‐septum toward the apical septum or right ventricular free wall. Such longitudinal deviation of the catheter tip may be difficult to detect in the LAO view. Another explanation relates to heterogeneity in α‐loop morphology. In the present study, the α‐loop was visually defined in the LAO view as any catheter configuration resembling the Greek letter “α,” without detailed geometric or quantitative characterization. Variations in loop size, curvature, or orientation may therefore have influenced the final implantation site, leading to misclassification. These findings suggest that qualitative recognition of an α‐loop should be interpreted cautiously and in conjunction with other fluoroscopic views, such as the RAO view.

### 4.2 The Predictor of a Micra Implantation on the Ventricular Mid‐Septum Obtained During the Implantation Procedure

Mid‐septal Micra deployment is now considered the preferred approach for minimizing fatal cardiac injuries, even though implantations initially targeted the ventricular apex at launch [1, 3, 4, 17]. Accurate catheter tip positioning is crucial to prevent implantation at the septum/free wall boundary (hinge) [18, 19]. Studies have reported that fluoroscopy and CT scans can aid in secure implantation. Tachibana et al. highlighted preoperative CT as a tool for assessing the anatomical relationship between the inferior vena cava and right heart structures, thereby improving safety during catheter manipulation [4]. Hai et al. demonstrated that multi‐angle fluoroscopy (RAO, LAO, and left lateral views) resulted in successful mid‐septal implantation in 90% of cases [3]. Li et al. found that right ventriculography using RAO and LAO views with 20 cc of contrast media enhanced visualization of the ventricular septum for precise Micra placement [20]. Although various techniques have been developed to facilitate mid‐septal Micra implantation, identifying reliable intraoperative predictors remains challenging. In our study, the α‐loop shape of the delivery catheter—clearly visible under fluoroscopy—was associated with mid‐septal placement and may serve as a practical intraoperative indicator. While not all cases with an α‐loop shape appearance resulted in mid‐septal implantation, its presence showed a meaningful trend toward favorable positioning. This finding may be beneficial for patients with impaired renal function, as it does not require contrast media. Moreover, mid‐septal implantation enables narrow QRS pacing, potentially contributing to the prevention of pacemaker‐induced cardiomyopathy (PICM) [7, 21].

## 5 Limitations

Several limitations need to be acknowledged. Firstly, this is a single‐center, retrospective, observational design. Secondly, none of the patients underwent Micra implantation on the ventricular basal septum. Therefore, the relationship between the catheter shape and Micra implantation on the basal septum remains unclear. Thirdly, RAO‐derived parameters were not included as predictive factors in the study. Future studies incorporating multimodal imaging or quantitative RAO‐based metrics may further refine intraoperative prediction of mid‐septal Micra implantation. Finally, anatomical variation of the right heart and differences in operator technique may limit generalizability.

## 6 Conclusion

The α‐loop shape of the delivery catheter in the LAO fluoroscopic imaging could be a reliable predictor for successful Micra implantation on the ventricular mid‐septum via the femoral venous approach.

## Author Contributions

**Misun Pak:** conceptualization, data collection, analysis, and writing – original draft. **Ken Kakita:** supervision and final approval of the manuscript. **Takashi Yamasaki:** data collection. **Tetsuhisa Hattori:** critical revision of the manuscript. All authors have read and approved the final manuscript.

## Funding

The authors have nothing to report.

## Ethics Statement

This study protocol complied with the Declaration of Helsinki and was approved by the Research Ethics Committee of Koseikai Takeda Hospital (approval no. 2406; approval date: July 11, 2024). The requirement for written informed consent was waived. The ethics committee reached this recommendation because an observational design without prespecified interventions for the study patients was employed. However, the opt‐out option in the study protocol guarantees the right to reject enrollment. We have read and understood the journal's policies and believe that neither the manuscript nor the study violates them.

## Conflicts of Interest

The authors declare no conflicts of interest.

## Data Availability

The data that support the findings of this study are available from the corresponding author upon reasonable request.
